# Advances in magnetic induction hyperthermia

**DOI:** 10.3389/fbioe.2024.1432189

**Published:** 2024-08-05

**Authors:** Yun-Fei Zhang, Mai Lu

**Affiliations:** Key Laboratory of Opto-Electronic Technology and Intelligent Control of Ministry of Education, Lanzhou Jiaotong University, Lanzhou, China

**Keywords:** magnetic induction hyperthermia, magnetic heating materials, induction coil, biological experiment, clinical trial, numerical simulation

## Abstract

Magnetic induction hyperthermia (MIH), is a technique that has developed rapidly in recent years in the field of tumor thermotherapy. It implants a magnetic heating medium (millimeter-sized heat seeds, micron-sized magnetic particles and nanometer-sized magnetic fluids, etc.) inside the tumor. The material heats up under the induction of an external alternating magnetic field (100–500 kHz), which causes a high temperature zone to rapidly form in the local biological tissues and induces apoptosis in tumor cells. Magnetic induction hyperthermia has the advantages of high safety, strong targeting, repeatable treatment, and the size of the incision during treatment is negligible compared to surgical resection, and is currently used in clinical treatment. However, the millimeter-scale heat seed heating that is typically used in treatments can result in uneven temperatures within the tissue. Common MIH heating devices are bulky and complex in design, and are not easy for medical staff to get their hands on, which are issues that limit the diffusion of MIH. In this view, this paper will discuss the basic theoretical research on MIH and the progress of MIH-related technologies, with a focus on the latest research and development results and research hotspots of nanoscale ferromagnetic media and magnetic heat therapy devices, as well as the validation results and therapeutic efficacy of the new MIH technology on animal experiments and clinical trials. In this paper, it is found that induction heating using magnetic nanoparticles improves the uniformity of the temperature field, and the magneto-thermal properties of nanoscale ferromagnetic materials are significantly improved. The heating device was miniaturized to simplify the operation steps, while the focusing of the magnetic field was locally enhanced. However, there are fewer studies on the biotoxicity aspects of nanomedicines, and the localized alternating magnetic field uniformity used for heating and the safety of the alternating magnetic field after irradiation of the human body have not been sufficiently discussed. Ultimately, the purpose of this paper is to advance research related to magnetic induction thermotherapy that can be applied in clinical treatment.

## 1 Introduction

**FIGURE 1:**
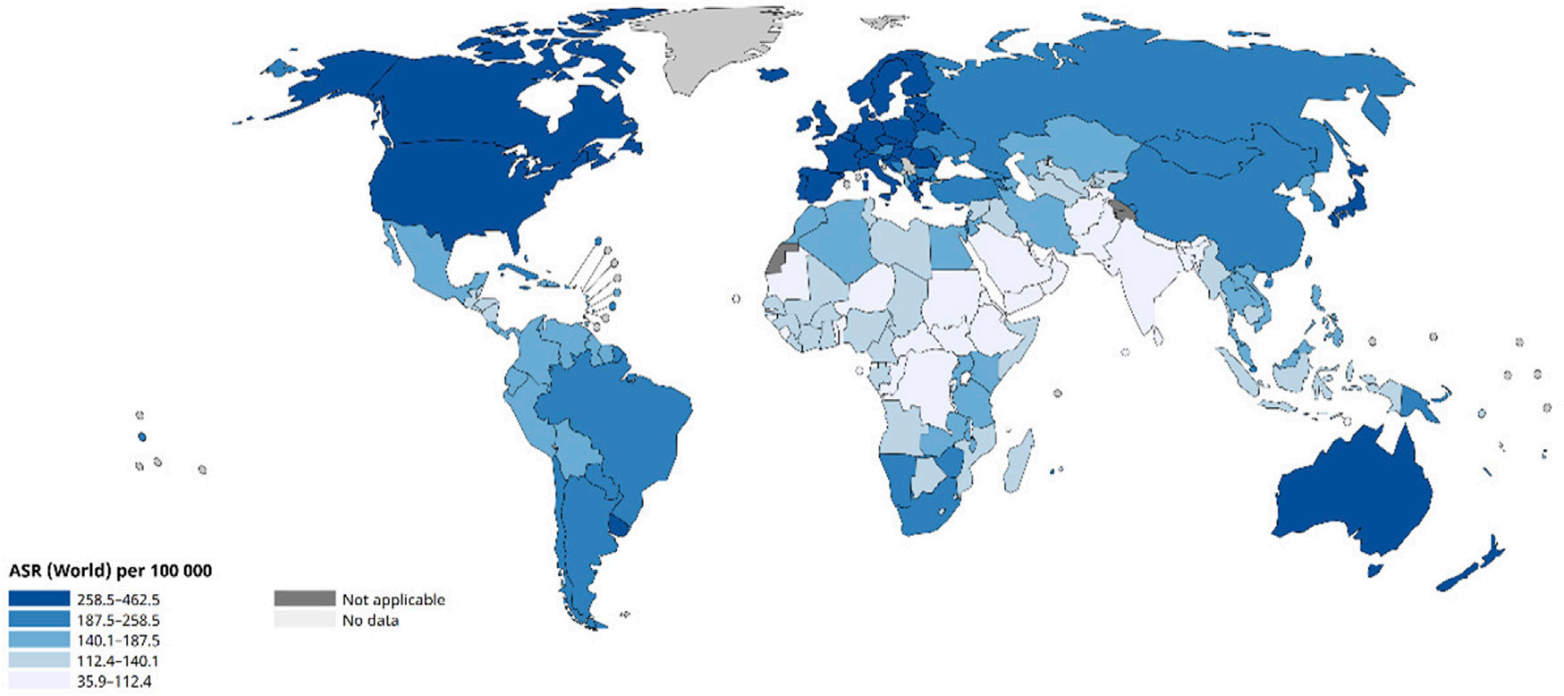
Age-Standardized Rate (World) per 100 000, Incidence, Both sexes, in 2022 (Ferlay et al., 2024).

Cancer is a generic term for a group of multiple diseases that can affect any part of the body, which is a serious public health challenge being faced all over the world, as shown in Figure 1. According to information released by the World Health Organization, in 2020, cancer had killed nearly 10 million people worldwide, a figure that accounts for about one-sixth of all global deaths that year (de Martel et al., 2020; World Health Organization, 2020; World Health Organization, 2022). Most cancers can be cured if detected and treated at an early stage.

Typically, cancer is treated with surgery, radiotherapy, and systemic therapies including chemotherapy, hormonal treatments, and targeted biological therapies. However, these methods are often accompanied by more obvious side effects. For example, surgical resection will leave wounds and may cause metastasis of cancer cells; chemotherapy uses chemicals that may have toxic side effects on normal cells in the body; and radiotherapy uses rays that may destroy normal cells under prolonged action.

In the last decades, exposing diseased tissues to higher temperatures has been considered for some time in the comprehensive treatment of tumors as an adjuvant therapy, which has low side effects (Falk and Issels, 2001; Van der Zee, 2002). Cells are susceptible to thermal damage because heat is indiscriminately disrupting the energy of cellular pathways, especially through its effects on protein structure and function (Needham et al., 2000; Zhang and Calderwood, 2011). Hyperthermia, is the use of different heat sources that cause cells to absorb a sufficient dose of heat. Elevated levels of heat shock proteins in tissues or cells are induced by high temperatures, which in turn cause a series of physiological changes that ultimately lead to apoptosis (Dewhirst et al., 2003).

Common methods of heating tumors rely on ultrasound (Wu et al., 2003; Furusawa et al., 2007; Bachu et al., 2021; Suarez-Castellanos et al., 2023), radiofrequency (Kok et al., 2015; Peeken et al., 2017; Chiao et al., 2022; Orlacchio et al., 2023), photothermal (Mura et al., 2013) and magnetic induction thermotherapy (Hedayatnasab et al., 2017). However, these methods have limitations while curing cancer. For example, ultrasound is difficult to address resorption and reflection by bones; The electromagnetic fields of radiofrequency are evanescent, making it difficult to achieve precise heating of the tumor area, which may cause medically induced thermal damage to nerves and tissues in the vicinity of the treatment target (Filippiadis et al., 2014); ultraviolet and visible light commonly used in photothermal therapy are absorbed by the chromosomes of biological tissues (mainly hemoglobin, myoglobin, and melanin), and the ability to penetrate tissues is poor, resulting in a usually shallow heating depth that makes it difficult to treat diseased tissues deep in human tissues (Wang and Kohane, 2017).

**FIGURE 2:**
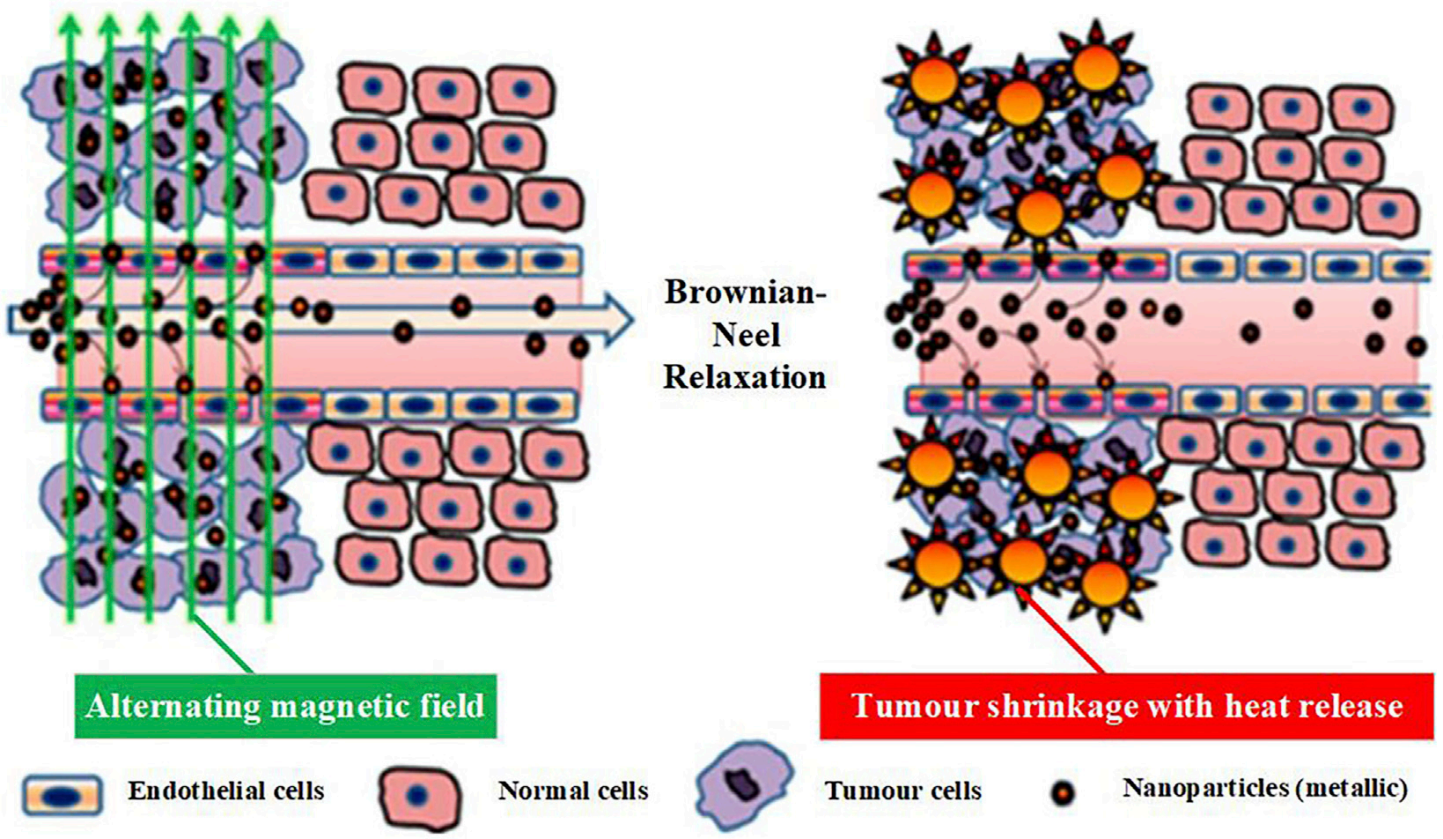
Schematic diagram of the principle of magnetic induction thermotherapy (Mahendravada et al., 2024).

As a means of heat therapy, magnetic induction thermotherapy has long been under close scrutiny by researchers. Typically, magnetic induction hyperthermia uses low-frequency (usually 50–400 kHz) alternating magnetic fields, shown in Figure 2.

In this frequency band, electromagnetic waves provide high penetration and pass through biological tissues with less attenuation and reflection. In addition, low frequency alternating magnetic fields produce negligible thermal effects from their interaction with tissues (Greenebaum and Barnes, 2006; Barnes and Greenebaum, 2018). In this paper, we will illustrate the research progress of magnetic induction hyperthermia from the aspects of basic research, simulation and calculation, medical application, and magnetic heating materials.

## 2 Fundamental principles research

Magnetic induction hyperthermia (MIH) was first proposed by Gilchrist et al. (1957) in 1957. It was developed as a tumor physiotherapy method by taking advantage of the poorer heat tolerance of tumor cells compared with normal cells. The principle is that the magnetic medium is placed in the tumor site, and under the action of the applied alternating magnetic field, the thermal medium absorbs the energy of the external magnetic field and converts it into thermal energy, which leads to the formation of high-temperature zones of the tumor tissues by rapid local warming, thus inducing apoptosis and necrosis of the tumor cells (Gordon et al., 1979; Jordan et al., 1996; Chung and Shih, 2014; Zhao et al., 2013). In addition to the direct destruction of target cells by high temperature, other therapeutic mechanisms identified so far include 1) high temperature regulates the activity of various types of immune cells, including antigen-presenting cells and T cells, and activates the anti-tumor immune system (Repasky et al., 2013; Liu et al., 2019); 2) sensitizes cancer cells to adjuvant treatments such as radiotherapy and chemotherapy (Maier-Hauff et al., 2011; Datta et al., 2015; Issels et al., 2018); and 3) induces the formation of more mature cell types in the surviving cancer cells, thereby inhibiting their self-renewal (Evans et al., 2015; Moise et al., 2018).

Active targeting strategies utilizing cancer specific ligands have emerged as a promised solution for the specific delivery of magnetic nanoparticles (MNPs) to tumor sites. These ligands facilitate the internalization of targeted MNPs through receptor-mediated endocytosis, enhancing their accumulation in cancer cells while minimizing uptake by healthy tissues. Meanwhile, active targeting of nanomedicine conjugates utilizes various bioreceptors, including human epidermal growth factor, transferrin, folate, luteinizing hormone-releasing hormone, integrins, CD20, CD44, CD95, vascular endothelial growth factor, CXCR4, etc., in order to facilitate their entry into the tumor site. Table 1 lists the different bioreceptors, which are coupled with magnetic nanoparticles to enhance the efficacy of cancer treatment under magneto-thermal effect (Srivastava N. et al., 2022).

**TABLE 1:** Magnetic nanoparticles conjugated with different biological receptors used in magnetic hyperthermia against different cancer cell lines.

Magnetic nanoparticle conjugates	Receptor/antigen	Cancer cell lines	Reference
Mn-Zn ferrite nanoparticles	Integrin	RAW264.7 macrophage cells, Murine mammary carcinoma cells & Human umbilical vein endothelial cells	Xie et al. (2016)
Superparamagnetic iron Oxide nanoparticles	CD44	Human and neck squamous cell carcinoma	Su et al. (2019)
Heat Shock Protein Inhibitor-loaded Silica-coated Fe_3_O_4_ nanoparticles	CD20	Lung cancer stem cells	Zhao et al. (2017); Liu et al. (2020a)
Magnetite-based nanoparticles	CD133	Colorectal cancer cells	Yang et al. (2020)
(PLGA)-coated Superparamagnetic Iron Oxide Nanoparticles	Folate receptor, transferrin receptor	Breast and glial cancer cells	Balasubramanian et al. (2014)
Dextran spermine magnetic nanoparticles	Human epithelial growth factor receptor2	Breast cancer cells	Srivastava et al. (2022b)
Polyethylene glycol-coated magnetic nanoparticles	Luteinizing hormone-releasing hormone	Ovarian cancer cells	Taratula et al. (2013)
Magnetite nanoparticles	CXCR4	Human acute T-cell leukemia	Vilas-Boas et al. (2018)
Zn^+2^ doped Superparamagnetic Iron Oxide Nanoparticles	Vascular endothelial growth factor	Liver cancer cells	Pan et al. (2021)

Thermal media can be classified according to the size mainly into millimeter-sized metal hot seeds, micron-sized magnetic microspheres and nanometer-sized magnetic nanoparticles, and different sizes of thermal media correspond to different heat production mechanisms.

Millimeter metal heat seeds are mainly some alloy materials. In an alternating magnetic field, the magnetic flux within the alloy material changes from moment to moment, producing induced currents. These induced currents flow in the form of eddies, and accordingly, a magnetic field is generated to weaken the change of the external magnetic field. At this point, the heating of the metallic material mainly originates from the energy loss resulting from the eddy currents overcoming the change in magnetic flux (Brezovich et al., 1984; Brezovich and Meredith, 1989).

**FIGURE 3:**
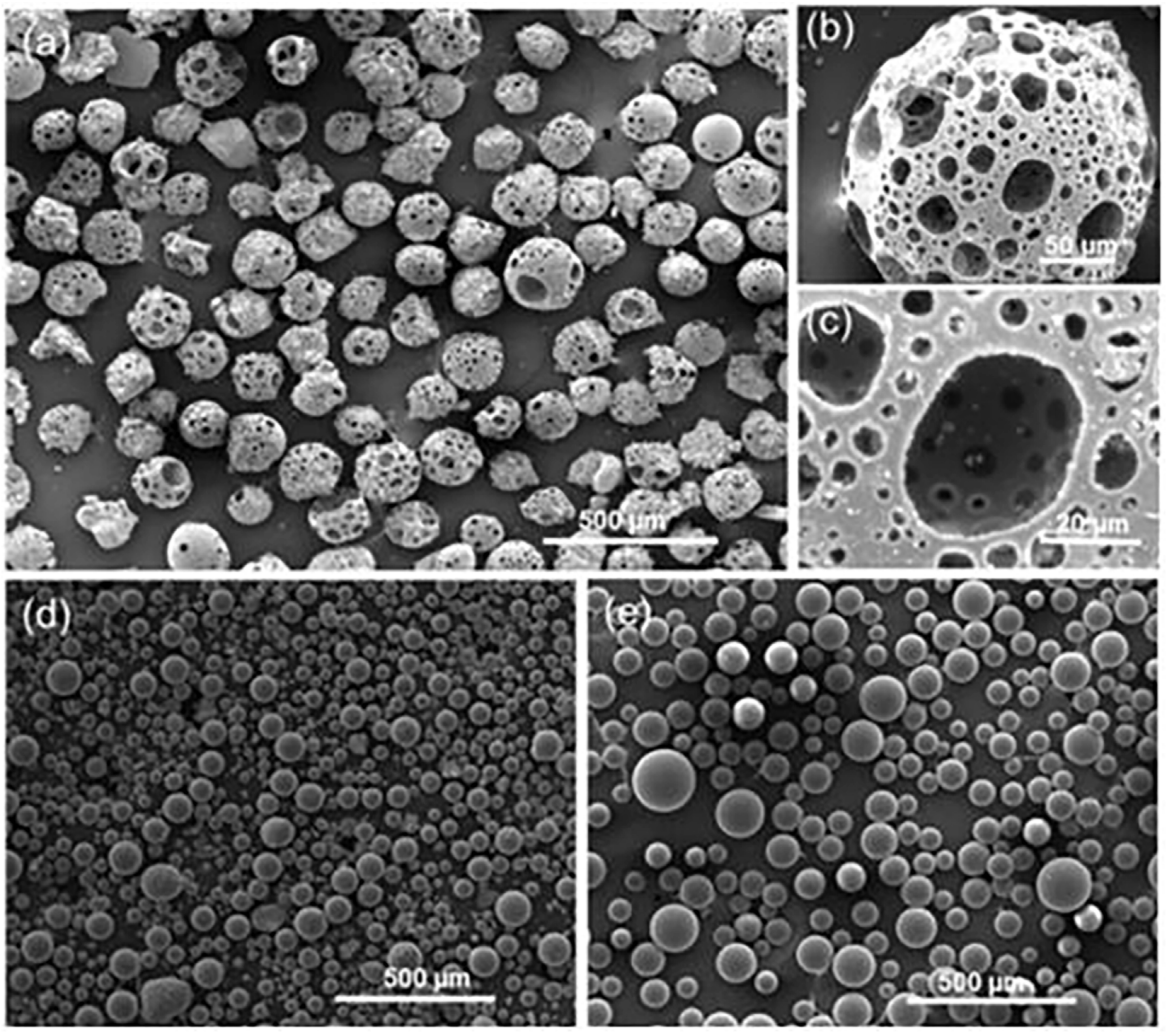
SE images of flame spheroidized **(A)** P_40_-Fe_3_O_4_ porous microspheres (125–212 µm); with **(B)** details of a porous microsphere; and **(C)** highlight of microsphere interconnected porosity. Flame spheroidized, unsieved. **(D)** Fe_3_O_4_ dense microspheres (≈20–100 µm), and **(E)** P_40_ dense microspheres (≈30–150 µm), used as controls (Molinar-Díaz et al., 2023).

Micron-scale media are mainly made of nanoscale particles polymerized (Miyagawa et al., 2014), as shown in Figure 3.

When in an alternating magnetic field, the magnetic domain and magnetic moments of materials change, resulting in the change of the magnetic induction strength inside the material is not synchronized with the change of the applied magnetic field, but lags behind the change of the magnetic field, i.e., the magnetic hysteresis. During quasi-static repeated magnetization, the energy loss due to the hysteresis effect is proportional to the area enclosed by the hysteresis loop (Pankhurst et al., 2003), as shown in the following equation.

$$P=\mu_{0}f\oint HdM$$

Where $\mu_{0}$ is the permeability of vacuum, the value is 4π × 10^−7^, and the unit is T⋅m/A. f is the magnetic field frequency, the unit is Hz. H is the magnetic field intensity, the unit is A/m. M is the magnetization, the unit is A/m.

**FIGURE 4:**
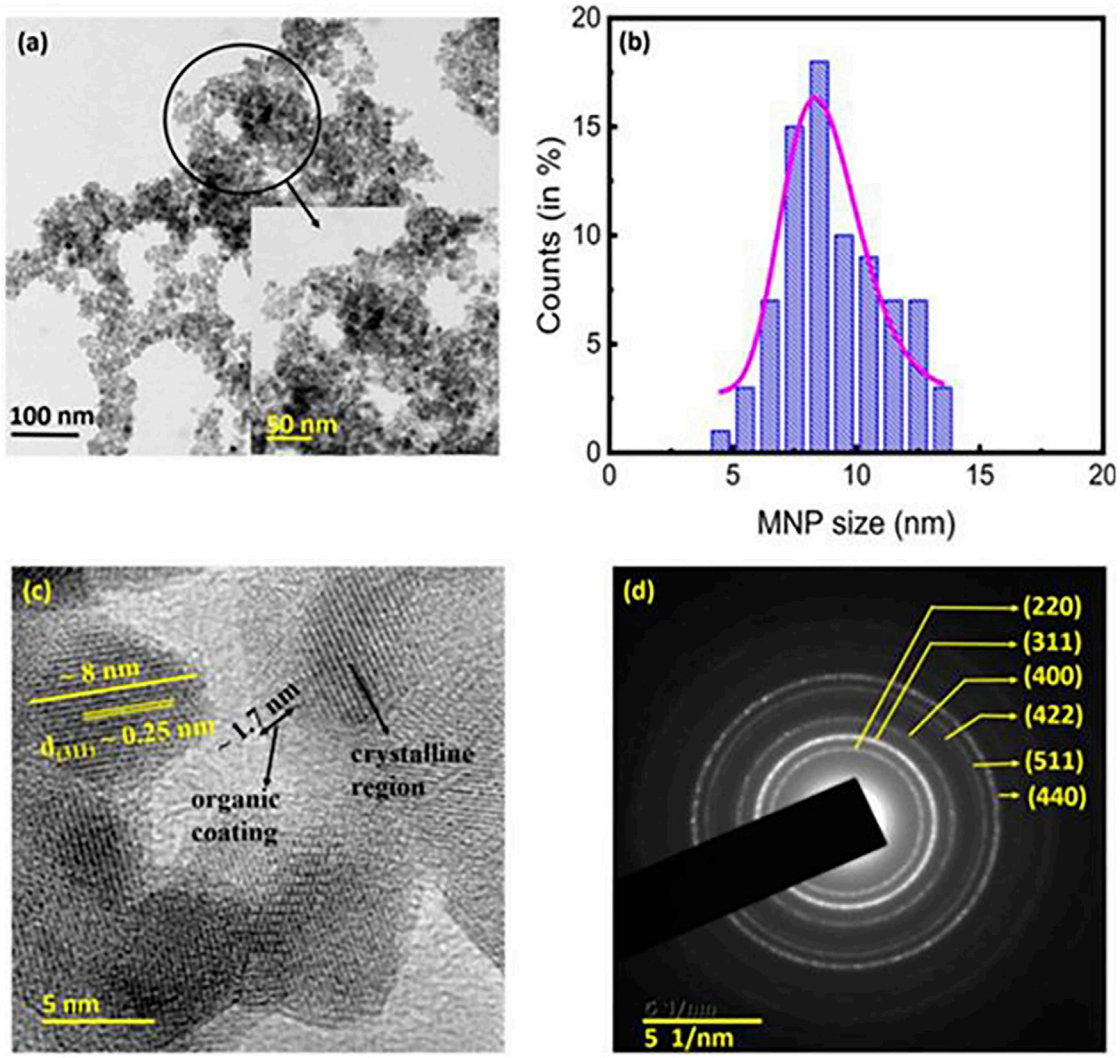
TEM image of the chitosan-coated Fe_3_O_4_ MNPs. **(A)** TEM image of the MNPs. (Inset) A magnified view of the encircled section of **(A)**. **(B)** Particle size distribution of the MNPs. The average crystallite size of ∼8.2 ± 0.9 nm obtained from the XRD analyses. **(C)** High-resolution transmission electron microscopy image of the MNPs. The presence of organic coating (average thickness ∼1.7 nm), and the d_(311)_ spacing of ∼0.25 nm were clearly discernible. **(D)** Selected area electron diffraction pattern showing the circular rings corresponding to the various crystallographic planes, which indicated good crystallinity of the prepared MNPs (Mahendravada et al., 2024).

Heat generation by relaxation is a mechanism used to characterize the heat generation of superparamagnetic singledomain particles under an alternating magnetic field. Figure 4 shows TEM image of a kind of nanoparticle. When the size of nanoscale particles is below a critical size (usually 10 nm), the single ancient nanoparticles will heat up due to the presence of Brownian relaxation loss and Neel relaxation loss (Delavari et al., 2013).

Among them, Brownian relaxation is the relaxation behavior resulting from the rotation of the magnetic material overcoming the thermal perturbation; Neel relaxation is the relaxation behavior resulting from the flipping of the magnetic moment inside the magnetic material overcoming the anisotropic properties. According to Rosensweig theory, the heating power of superparamagnetic nanomaterials can be expressed as follows (Rosensweig, 2002).

$$P=\mu_{0}\pi H^{2}\chi_{0}\frac{2\pi f\tau_{eff}}{1+{\left(2\pi f\tau_{eff}\right)}^{2}}$$

Where $\chi_{0}$ is magnetic susceptibility. $\tau_{eff}$ is relaxation time, the unit is s.

**FIGURE 5:**
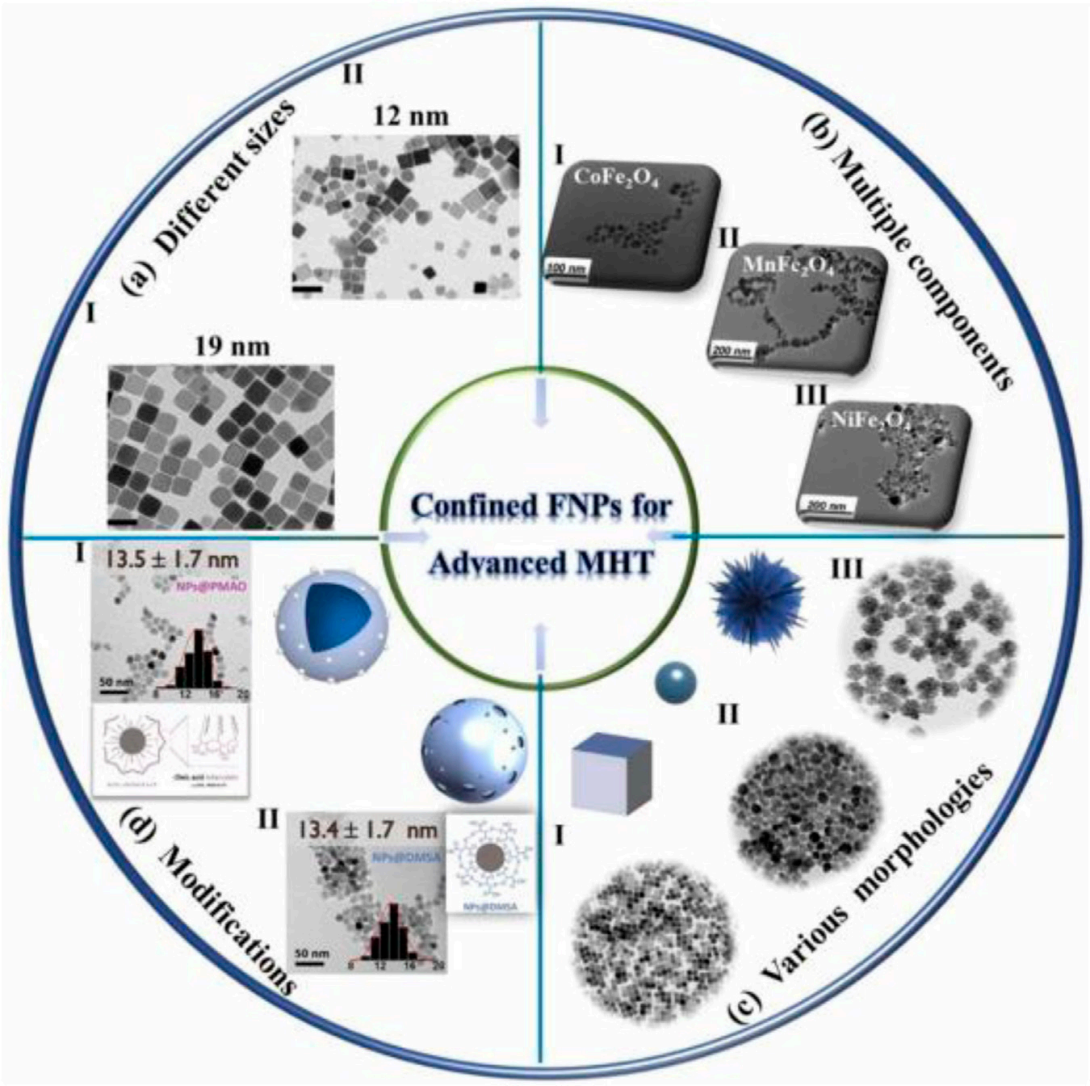
Influence factors on the heat generated of MIH, including size, component, morphology and surface modification (Zhang et al., 2024).

Extensive research has been conducted over a significant period to advance the use of magnetic nanoparticles in MIH. The thermal properties of magnetic nanoparticles (MNPs) are related to their own size, morphology, composition, anisotropy, and surface modifications. Influence factors on the heat generated of MIH are shown in Figure 5.

Lv et al. (2015) synthesized octahedral Fe_3_O_4_ nanoparticles of different sizes and shapes and investigated their heating properties. Octahedral nanoparticles of different sizes (13, 22, 43, 98 and 260 nm) and shapes were obtained. The SAR values of the samples were tested in alternating magnetic fields of certain frequency and different amplitudes. At the same magnetic field amplitude, the larger size (98 nm) of Fe_3_O_4_ has a higher SAR value (2629 W/g) compared to the smaller size of Fe_3_O_4_ (43 nm, 2483 W/g). Therefore, the size of the sample is an important factor affecting the heat production by MIH. Pardo et al. (2020) synthesized magnetic nanoparticles doped with different transition metal elements Mn^2+^, Co^2+^, and Zn^2+^. Within 15 min, the specific absorption of Zn_0.25_Co_0.29_Mn_0.21_Fe_2.25_O_4_ was up to 97 W/g (850 KHz), which is about three times higher than that of pure Fe_3_O_4_ (37.3 W/g). Therefore, the heat production can be controlled by adjusting the dopant metal.

Numerous nanoplatforms based on various morphologies of MNPs have been developed with the aim of effective cancer therapy. Without compromising the intrinsic properties of the Without compromising the intrinsic properties of MNPs, combining them with polymer-organic compounds can provide novel composites with high performance for biomedical applications. MNPs for biomedical applications are usually superparamagnetic nanoparticles with a radius of less than 20 nm, but their small size and weak magnetic properties lead to low magneto-thermal conversion efficiency (Wang et al., 2015). The SAR of the nanoparticles was greatly improved by adjusting the K value by forming a core-shell structure of soft and hard magnetic materials. CoFe_2_O_4_@MnFe_2_O_4_ has a high specific power loss of 2,280 W/g, much higher than that of 9 nm CoFe_2_O_4_ (443 W/g) and 15 nm CoFe_2_O_4_ (411 W/g) (Sabale et al., 2019). Optimizing the physical properties of MNPs offers excellent prospects for better biocompatibility and dispersion, leading to superior magnetic hyperthermia performance.

The thermal effects of MNPs are also closely related to the surface modification layer. The introduction of appropriate coatings on MNPs can lead to prolonged blood circulation, good biocompatibility and further functionalization. The addition of a surface modification layer can significantly affect the thermophysical properties of MNPs, which in turn enhances the effect of magnetic thermotherapy (Li et al., 2022). Due to the biocompatibility and dispersion of MNPs, modified magnetic nanoparticles have been used in MIH over unmodified magnetic nanoparticles. However, it is important to note that the MNPs on the coating should be kept thin to avoid decreasing M_s_ and SAR values (Jamir et al., 2023).

Anandhi et al. (2020) used infrared thermography to study the AC magnetic heating mechanism associated with magnetic anisotropy at low Co doping in Fe_3_O_4_. It was found that the magnetic anisotropy constants of Fe_3_O_4_ and Co-doped Fe_3_O_4_ nanoparticles with an average particle size of 10–12 nm were increased from 16 to 31 KJm^–3^, respectively. Effective specific absorption rate (ESAR) of the nanoparticles was determined by infrared thermography and compared. The ESAR value of Fe_3_O_4_ nanoparticles were found to be 3.16 nHm^2^kg^–1^ and Co doping decreased the ESAR value to 2.84 nHm^2^kg^–1^. Reducing the dipole interaction strength overcomes the problem of heating of Stoner-Wohlfarth particles of reduced size. Surface-modified particles have low magnetic anisotropy for better heating efficiency. Lemine et al. (2023) conducted a systematic study of the structural, DC and AC magnetic properties of γ-Fe_2_O_3_, γ-Fe_2_O_3_ (Gd-5%) and γ-Fe_2_O_3_ (Co-5%) nanoparticles. AC magnetic measurements showed that γ-Fe_2_O_3_ (Co-5%) has a higher heating efficiency and SAR value (120 W/g), which is six times higher than that of γ-Fe_2_O_3_ or γ-Fe_2_O_3_ (Gd-5%) nanoparticles. This is mainly attributed to their higher effective anisotropy and saturation magnetization strength, where the thermal dissipation of γ-Fe_2_O_3_ (Co-5%) nanoparticles is mainly dominated by hysteresis loss, whereas that of γ-Fe_2_O_3_ or γ-Fe_2_O_3_ (Gd-5%) nanoparticles is mainly induced by Neel relaxation loss.

Magnetic induction thermotherapy can be subdivided into warm (42°C–46°C), high temperature (46°C–70°C), and thermal excision (70°C–90°C). In general, normal cells have better thermal tolerance compared to tumor cells, and irreversible thermal damage to tumor cells can be caused by temperatures above 42°C, whereas temperatures above 46°C cause irreversible thermal damage to normal cells. Therefore, the temperature used in magnetic induction hyperthermia is usually between 42°C and 46°C. Among other things, warm heat treatment can cause apoptosis of cancer cells without essentially damaging healthy tissues, and it does not cause complications such as inflammation because cancer cells are actively apoptotic during this treatment (Liu X. et al., 2020).

In order to describe the temperature increase and heat transfer behavior of biological tissues induced by the induction of an alternating magnetic field in thermotherapy, the process can be characterized by solving Pennes’s bioheat transfer equation (Lv et al., 2005). In 1948, Harry H. Pennes, while analyzing the temperature of tissues of the forearm and arterial blood of a resting human, proposed for the first time a macroscopic-scale model of the classical bioheat transfer, which is also known as Pennes’s biological heat transfer equation (Pennes, 1948; Wu et al., 2015), as shown in the following equation

$$\rho_{i}c_{i}\frac{\partial T_{i}}{\partial x}=k_{i}\nabla^{2}T_{i}+\omega_{b}\rho_{b}c_{b}\left(T_{b}-T_{i}\right)+Q_{met}+P$$

Where $T_{b}$ and $T_{i}$ are the tissue and blood temperatures, the unit is K. $\rho_{i}$ and $\rho_{b}$ are the density of tissue and blood, the unit is kg/m^3^. $c_{i}$ and $c_{b}$ are the specific heat capacity of tissue and blood, the unit is J/(kg∙K). $k_{i}$ and $\omega_{b}$ represent the specific thermal conductivity of tissue and local blood perfusion rate, the unit is kg/(m^3^∙s). $Q_{met}$ represents the local metabolic heat production per unit volume and is called metabolic heat, the unit is J. P is the heat production outside of biological tissues, the unit is J.

The model simplifies the actual organism while effectively revealing the basic laws of heat exchange within the organism’s tissues. In addition, the flow of blood in blood vessels, external heat sources, metabolism and the influence of various factors in the heat transfer process in the organism are comprehensively considered. The Pennes model has also been widely recognized by researchers in this field for its simplicity and validity, and some scholars have proposed an improved model based on it, which is closer to the real situation for the description of heat transfer in local biological tissues (Brown, 1965; Jiji et al., 1984; Weinbaum et al., 1984).

The complexity of the human physiological structure and the interaction of the organism’s environment with the magnetic thermal medium are difficult to solve by analytical methods. Numerical methods in the modeling of heat transfer phenomena are an effective way to solve this complex problem. Most of the simulations of heat transfer in magnetically induced thermotherapy are performed using the finite element method (FEM) or finite difference method (FDM).

Tang et al. (2023a) improved the uniformity of the magnetic field by adding a third coil between two in the conventional Helmholtz coils based on the finite element method of COMSOL software modeling calculations. A differential evolutionary algorithm was used to optimize the parameters of the improved coil. The improved coil showed a significant improvement in the uniformity of the magnetic field compared to the conventional coil. In terms of treatment efficiency, the improved coil can obtain better thermotherapy temperature distribution in the tumor region, and the advantage is more obvious when the tumor is far away from the center of the coil. Tang et al. (2023b) proposed a control method for the magnetic induction thermotherapy system. The finite element method was used to solve the improved Pennes biological heat transfer equation to obtain the temperature field distribution of biological tissues.

The temperature of the tumor during magnetic induction hyperthermia is controlled by optimizing the parameters of the proportional integral differential (PID). Simulation results show that the control system can effectively regulate the power consumption of the MNP and furthermore accurately regulate the transient temperature inside the tumor. In addition, the system can automatically adapt to different situations during treatment.

Reis et al. (2016) investigated the application of the finite difference method for numerical simulation of the heat transfer process in magnetic fluid thermotherapy. Their model considered tissue metabolic heat and heat dissipation caused by blood vessels and designed algorithms to solve the heat transfer equations. To improve the performance of the model, they utilized parallel computing to increase the processing speed. The experimental results demonstrated the parallelized computation is very effective in improving the performance, yielding up to 242 times gain compared to sequential execution time.

Lodi et al. (2021) evaluated the effect of the distribution of magnetic nanoparticles on the scaffold on the quality of thermotherapy treatment of bone tumors. They determined the magnetic properties of the magnetic bone scaffolds and the attachment pattern of the nanomaterials, and performed multiphysics field numerical simulations of magnetically induced thermotherapy by multiphysics field simulations. The results showed that the SAR of the magnetic bone scaffold does not harm any non-target tissues. When magnetic nanoparticles were symmetrically placed on the scaffold, the temperature distribution along different axial directions had different trends. Kandala et al. (2021) reported a coupled electromagnetic and thermal model that can be used to estimate the temperature induced by eddy current heating in a uniform tissue model. The validated model was successfully used in the temperature analysis of a complex geometry of a rabbit liver tumor during magnetic induction thermotherapy.

## 3 Magnetic materials discovery and development

The development of magnetic materials is one of the keys to the development of magnetic induction thermotherapy technology. Current researchers are focus on the development of magnetic nanoparticles.

**FIGURE 6:**
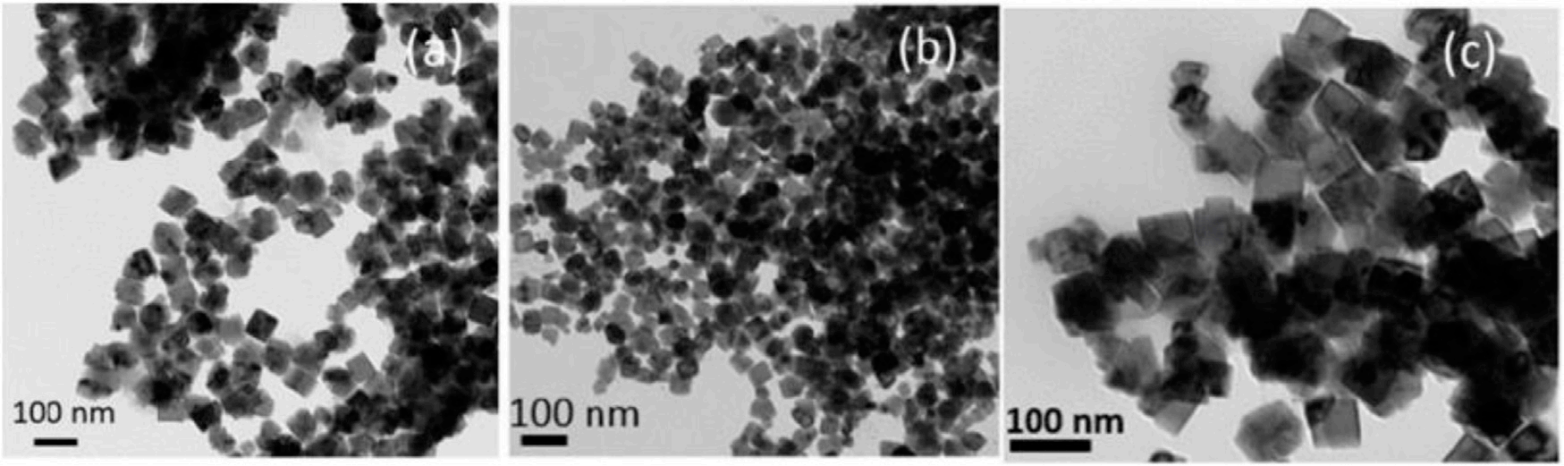
Transmission electron micrograph of the FeCo alloy nanoparticles synthesized at 90°C using the SMMP. **(A)** Without surfactant. **(B)** With PVP polymer. **(C)** With oleic acid and oleyl amine as surfactants (Krishnakumar and Harris, 2021).

Krishnakumar and Harris (2021) synthesized an alloy nanoparticle of FeCo, shown in Figure 6. The particles have a body-centered cubic (BCC) crystal structure with an effective particle size of 25–75 nm. The maximum saturated magnetization intensity of the sample synthesized at 90°C is 207 emu/g at room temperature and the magnetization strength is 100 Oe, which can be applied for the preparation of conjugated nanoparticles for diagnostic and therapeutic biomedical applications.

Tonthat et al. (2023b) presented the synthesis and characterization of ultrasmall iron oxide/gold composite nanoparticles (Fe_3_O_4_@Au NPs). Experimentally (Tonthat et al., 2023a), the team investigated the rapid heating of Fe_3_O_4_@Au nanoparticles and Fe_3_O_4_ nanoparticles using the ferromagnetic resonance (FMR) effect, comparing them with the results obtained under magnetic fields commonly used in conventional magnetic induction thermotherapy. The results show that the maximum value of the Fe_3_O_4_@Au nanoparticle heating rate increases with RF field frequency and that the value used to measure the initial rate of temperature rise is two orders of magnitude higher than in conventional magnetic induction thermotherapy. Bao et al. (2021) developed a new magnetite (denoted as nanoring Fe_3_O_4_ @PPy-PEG). The magnetite showed excellent thermotherapeutic efficacy when simultaneously exposed to an alternating magnetic field (300 kHz, 45 A) and a near-infrared (808 nm, 1 W cm^−2^) laser.

**FIGURE 7:**
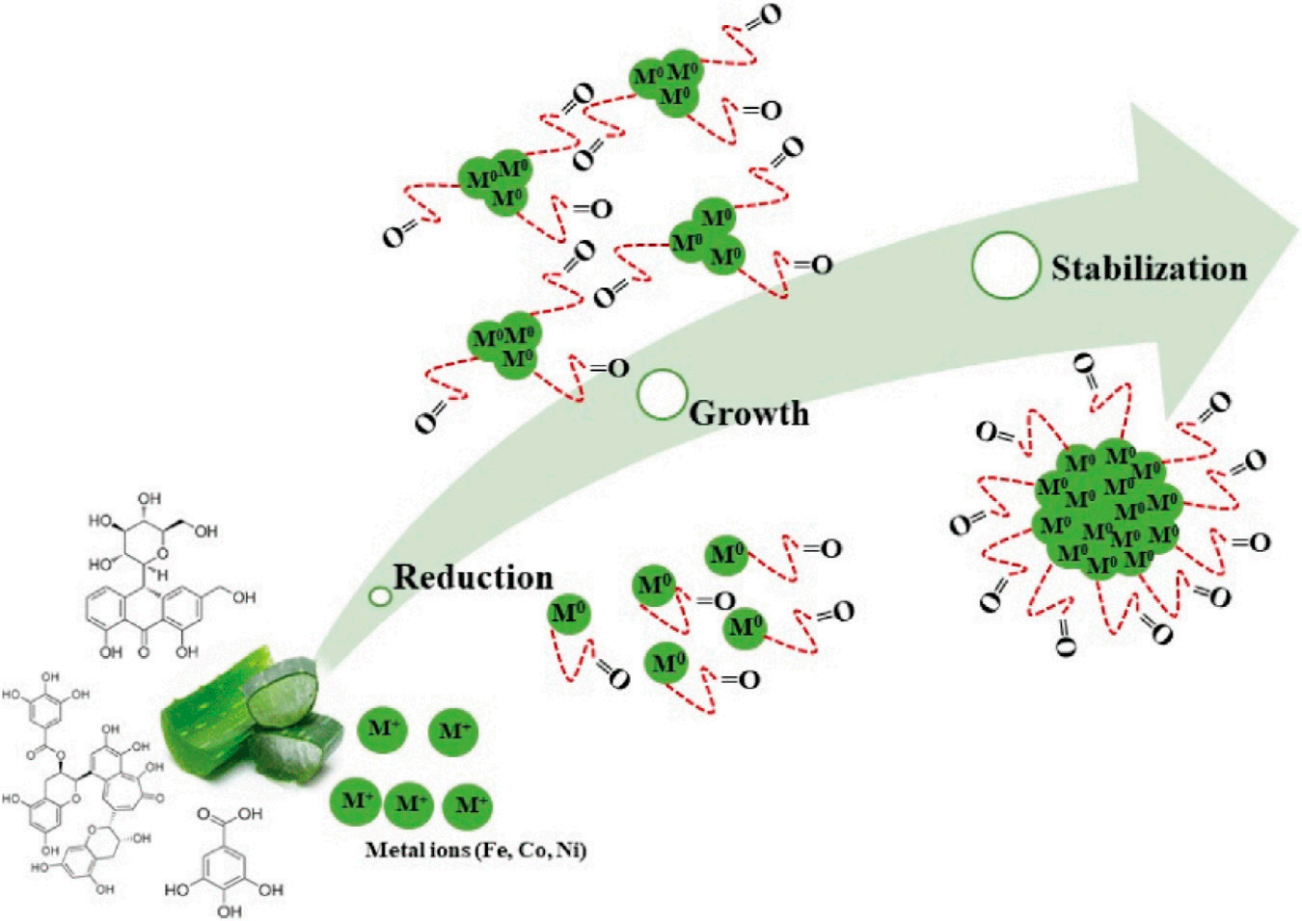
Mechanism formation of ferrites by plant leaf extract (Hermosa et al., 2022).

Iron oxides with MFe_2_O_4_ passages are also studied, where M can be elements such as Fe, Co, Al, etc. Srivastava M. et al. (2022) prepared a sample of trivalent Al ^3+^ doped magnetite (AlxFe_3_-_x_O_4_, 0. 01 ≤ x ≤ 1. 0) by wet chemical method. The sample had a spherical morphology with a size range between 4 and 25 nm. They found that the saturation magnetization intensity of the sample increased with increasing Al concentration during magnetothermal therapy, reaching a maximum value (1062 A-m2/kg) at x = 0.1. Thereafter, the saturation magnetization intensity began to decrease with increasing Al concentration. Hermosa et al. (2022) synthesized nano ferrite magnetic materials (Fe_3_O_4_, CoFe_2_O_4_ and NiFe_2_O_4_) using aloe vera extract as raw material by hydrothermal method. Mechanism formation of ferrites by plant leaf extract is shown in Figure 7.

The spherical nano ferrite magnetic materials’ diameters were in the size range of 6–28 nm and also exhibited high crystallinity. X-ray diffraction analysis also showed that the synthesized metal nanoparticles were all magnetic with saturation values of Fe = 52 emu/g, Co = 33 emu/g, and Ni = 20.43 emu/g. The nano ferrite magnetic materials showed high cell viability at high concentrations, biocompatibility good and non-toxicity.

Nguyen et al. (2021) synthesized MnFe_2_O_4_ nanoparticles with different particle sizes ranging from 11 to 70 nm using a hydrothermal method. Under a magnetic field with an amplitude of 80 Oe and a frequency of 236 kHz, they found that MnFe_2_O_4_ nanoparticles of 18 nm had the highest SAR of 65.52 W/g. This study provides a basis for accurately predicting the optimal size of magnetic nanoparticles in induction heating.

Researchers have also proposed other thermal media that can be used for magnetic induction thermotherapy. Janus MMSNPs have high magnetic response properties and magnetotherapeutic conversion efficiency. Zuo et al. (2022) investigated their magnetic targeting properties *in vitro* and *in vivo*. The results demonstrated favorable therapeutic outcome results and biosafety. Gao et al. (2019) developed a new adaptive magnetic hydrogel using ferromagnetic vortex-domain iron oxide nanorings (FVIOs) incorporated into chitosan-based dynamic hydrogels. The magneto-thermal properties of this hydrogel have significant advantages over superparamag-netic iron oxide nanoparticles (SPIOs). When heated to the same temperature, the concentration of FVIO (0.6 mg mL^−1^) required was nearly 17 times lower than that of SPIO (10 mg mL^−1^). *In vivo* studies have shown that the adaptive magnetic hydrogel therapy system developed by the team contributes to combined chemo-thermal therapy. It can effectively destroy residual malignant tumor cells and prevent breast cancer recurrence, and is expected to be widely used in comprehensive cancer treatment.

Molinar-Díaz et al. (2023) developed a ferromagnetic glass-ceramic with a porous structure and good biocompatibility. When heated by magnetic induction, the P40- Fe_3_O_4_ microspheres exhibited a highly controlled heating profile, which eventually stabilized at 41.9°C, making them well suited for magnetic induction thermotherapy applications. Liang et al. (2023) developed a magnetic mSiO2-SmCox nanoparticle. They heated it and demonstrated the potential of the material to treat tumors due to magnetic and photothermal effects. Dong et al. (2020) co-loaded calcium peroxide (CaO_2_) and iron oxide (Fe_3_O_4_) nanoparticles into three-dimensionally (3D) printed magnesium xanthite feldspar scaffold (AKT- Fe_3_O_4_- CaO_2_) to accelerate osteosarcoma treatment. Self-supply of H_2_O_2_ can be achieved by synergistically inducing Fenton-like chemical reaction through magneto-thermal therapy with Fe_3_O_4_ nanoparticles producing heat under alternating magnetic fields, which can enhance the tumor treatment effect.

## 4 Instrument development

Researchers have developed magnetic field generators that can be used for *in vitro* biological tissue experiments, animal experiments, and clinical trials. Among them, Jordan’s group (Jordan et al., 1999) has made a series of breakthroughs that are at the forefront of the world.

**FIGURE 8:**
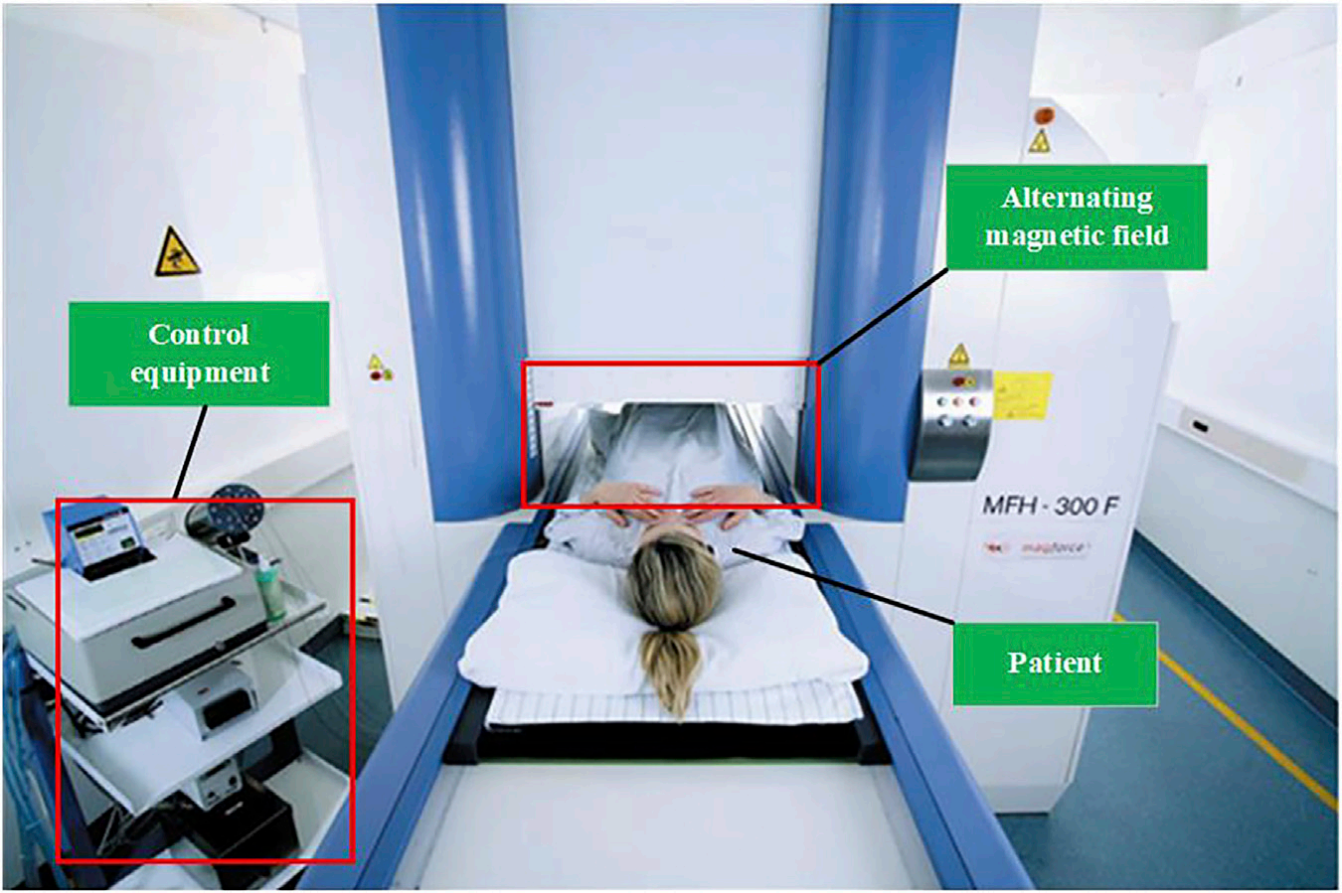
Clinical treatment with the MFH 300F (MagForce Nanotechnologies AG, Berlin, Germany) (Thiesen and Jordan, 2008).

In 1999, Jordan’s team (Jordan et al., 2001) developed a frequency- and amplitude-adjustable magnetic induction hyperthermia device with a magnetic field strength of 0–15 kA/m and an alternating frequency of 100 kHz base on their previous research. In 2004, Jordan’s team (Gneveckow et al., 2004), in conjunction with Magforce, developed a prototype of the MFH 300F oncology magnetic induction hyperthermia machine prototype. This device is capable of applying an alternating magnetic field of 100 kHz, and the treatment area is a cylindrical area with a diameter of 20 cm and a height of 300 mm, with a magnetic field strength ranging from 12 to 18 kA/m. Clinical treatment with the MFH 300 F is shown in Figure 8. It has been applied in clinical treatment (Kobayashi et al., 2014; Harris et al., 2017; Grauer et al., 2019) with good results.

To address the problem of effectively implanting magneto-thermal media into tumors, Tonthat et al. (2020) developed a simple, fast, and low-cost automated positioning system. The system uses three pickup coils mounted symmetrically inside a drive coil and a robotic arm that can automatically operate based on the voltages induced in the three pickup coils to perform fine-tuned scanning (rotary scanning) and fine-tuned scanning (linear scanning) with an accuracy of within 1 mm.

Hadadian et al. (2020) combined images of magnetic nanoparticle and thermal ultrasound imaging with magnetic induction hyperthermia. They used an ultrasound device to capture images of magnetic nanoparticle and used ultrasound thermometry to monitor temperature changes during magnetic thermotherapy. The results showed that these magnetic nanoparticles were effectively used as contrast agents and generated heat during magnetic induction hyperthermia. Real-time two-dimensional temperature maps were obtained, and the results were in general agreement with measurements using a fiber-optic thermometer. Le et al. (2020) proposed a novel electromagnetic navigation and heating system that allows real-time imaging feedback to guide and navigate magnetic nanoparticles. Focused magnetic nanoparticle heating can also be used to release drugs while monitoring temperature. The team maximized the magnetic gradient and magnetic field within a field of view of ×30 30 mm. And based on this, a 2-D magnetic nanoparticle navigation/heating system was realized *in vitro*, providing a highly valuable diagnostic solution for magnetic induction hyperthermia.

**FIGURE 9:**
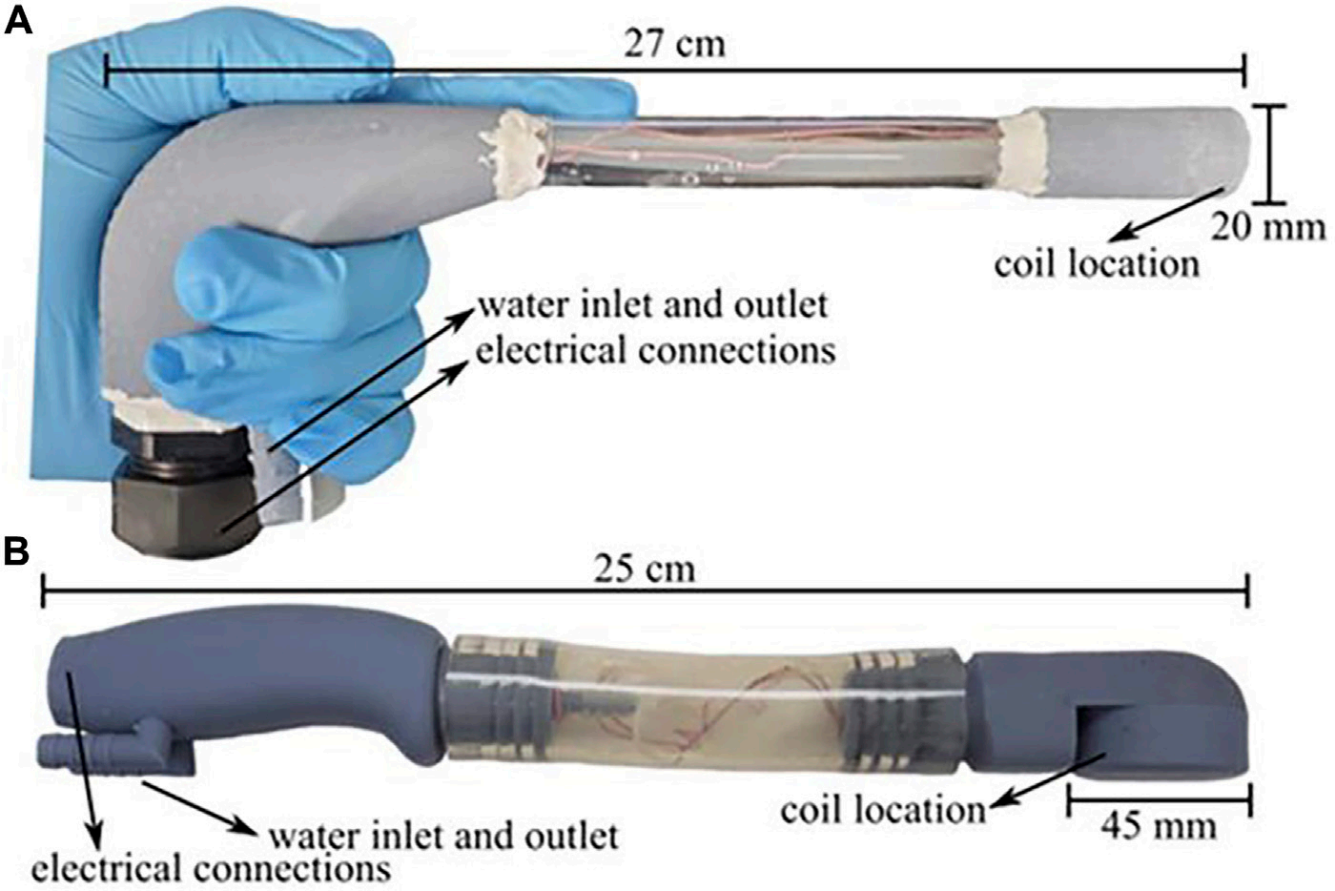
Laparoscopic and transrectal induction heater enclosures. **(A)** Laparoscopic induction heater and **(B)** Transrectal induction heater with lengths and diameter of the coil location shown. Both instruments were built with 3D printed parts and commercially available products (Castro-Torres et al., 2023).

The aim of magnetic induction thermotherapy is to selectively heat the tumor region, but existing studies have often placed an entire region of biological tissue in a high-frequency alternating magnetic field, which may cause nonessential biological effects. Therefore, Castro-Torres et al. (2023) proposed the design of two novel localized heaters, shown in Figure 9 and performed cellular experiments on both protocols to assess feasibility. Magnetic hyperthermia using both devices was found to kill cancer cells with minimal effect on normal cells, and the study has the potential to be complementary to existing cancer treatments.

As of now, the device developed by Jordan et al. has been approved for clinical use in the treatment of recurrent glioblastoma. However, we note that the device’s large size results in its poor mobility. Moreover, the device is unable to focus the magnetic field within a certain area, resulting in the therapeutic effect of MIH relying on the distribution of MNPs within the tumor. Hadadian and Le’s study of localized MIH assisted by medical imaging provides a better way of thinking for researchers. In the future, improving the local focus of the magnetic field, active targeting technology combined with bioimaging technology, and enhancing the portability of the device will be the focus of MIH device development.

## 5 Biological experiment

**FIGURE 10:**
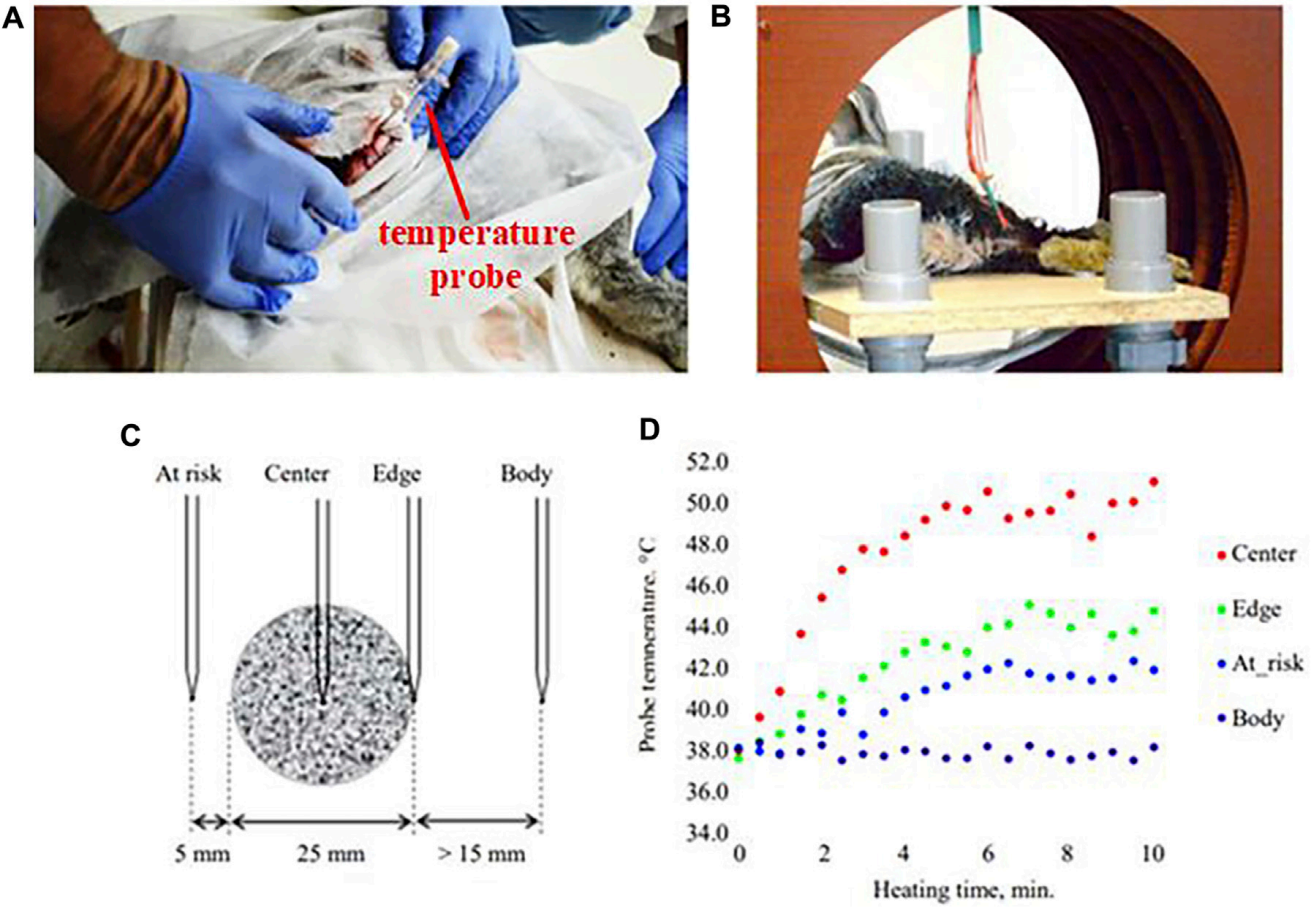
Results of the *in vivo* experiment: **(A)** implantation of an implant with catheters for temperature monitoring into a rabbit thigh; **(B)** placement of the rabbit in an inductor coil; **(C)** location of thermocouples; **(D)** heating kinetics (Vasilchenko et al., 2017).

Biological experiments are very important prior to clinical trials. In 1959, Medal et al. (1959) implanted magnetic particles into an inguinal lymphatic model in rabbits and heated them using helical coils. For the first time, it was demonstrated that selective placement of metal heat seeds in the tumor region under the irradiation of an alternating magnetic field could achieve localized hyperthermia of the tumor. Vasilchenko et al. (2017) used a prototype induction heating system to generate an alternating magnetic field with a frequency of about 100 kHz and a maximum intensity of up to 3 kA/m in an induction coil with an inner diameter of 35 cm. Heating experiments were carried out on a 2.5 cm diameter spherical implant *in vitro* and inside the thigh of a living rabbit, and in both cases the experimental results were in agreement with theoretical estimates. Measurements were found to produce effective hyperthermia at distances greater than 5 mm from the implant surface, and thermocouple contacts farther away from the implant surface (17 ± 1 mm) showed temperatures identical to the body temperature of rabbits, confirming that normal body tissues are not heated in an alternating magnetic field with a frequency of 100 kHz. The results of the *in vivo* experiment are shown in Figure 10.

Ouyang et al. (2010) implanted heat seeds into rat mammary tumors while the control group was left untreated. The experimenters found that necrosis and apoptosis of tumor cells could be observed in all experimental group rats after 14 days after the application of an external magnetic field. While the control group’s tumors grew to approximately 600% of their original volume, the experimental group’s tumors decreased in size by approximately 20% from the untreated group.

Sharma et al. (2023) proposed an automated device that can control tissue heating based on real-time temperature input measured by sensors. The team constructed a 20 cm diameter Maxwell induction coil powered by a 120 kW induction heating power supply that generates an alternating current of 160 kHz. In addition, the team performed *in vivo* validation of the heating effect in gel body molds and bovine liver slices. This was done by injecting magnetic nanoparticles (MNPs) into the brain of a canine research subject and heating it to see the results. The device could reach a user-selected target temperature within 60 s and maintain the user-selected target temperature for 15–30 min with a steady state error of <1%. Zhang et al. (2023) prepared PEI-Fe 3 O 4/pYr-ads-8- 5 HRE-cfosp-IFNG albumin nanospheres. *In vitro* heating experiments were performed on magnetic albumin nanospheres in an alternating magnetic field. Through animal experiments, gene therapy combined with magnetic fluid hyperthermia has shown promising results in both cellular and animal studies.

**FIGURE 11:**
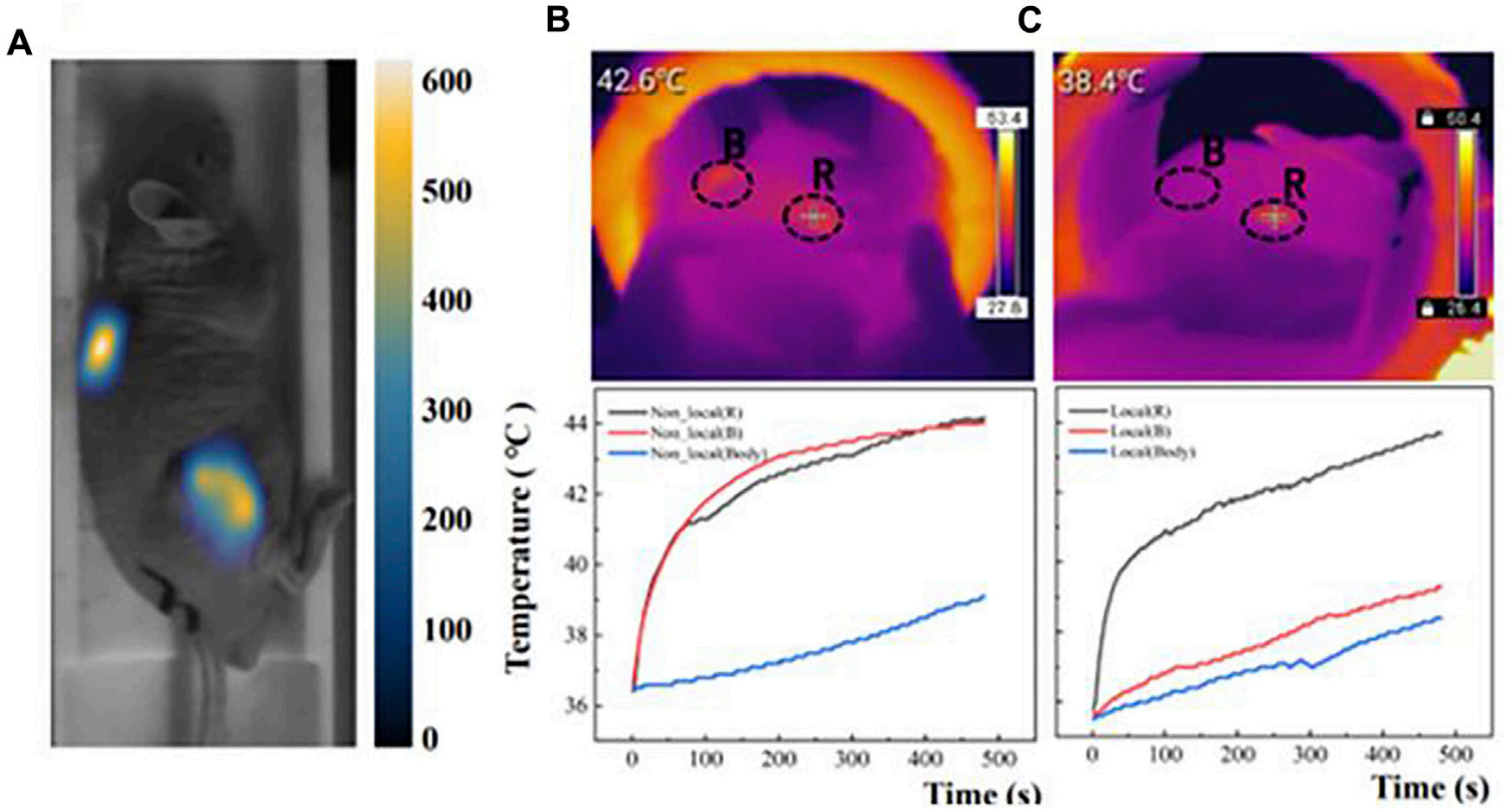
**(A)** MPI images of double-tumor after injection of 2 mg/mL Synomag-70. **(B)** Two tumors and body temperature changes during MFH in the non-localized group. **(C)** Two tumors and body temperature changes during MFH in the localized group (Lei et al., 2024).

Lei et al. (2024) developed a high gradient field MPI-MFH method for precise localized heating, which can achieve millimeterscale 3D localization and effective heating of low concentration regions, and validated the method by body model experiments and rat *in vivo* glioma model experiments, which are shown in Figure 11. The results showed that using MPI (Magnetic Particle Imaging) pixels as a guide for MFH (Magnetic Fluid Hyperthermia) parameters improved the MNP concentration gradient sensitivity to ±1 mg/mL. The method effectively inhibited the growth of targeted gliomas by localized heating, which allowed for more efficient heating without damaging normal tissues by heating the lesion more efficiently, and it is expected to achieve similar results in the treatment of other types of cancer.

Shirvalilou et al. (2023) proposed a magnetic induction hyperthermia combined with chemotherapy, which was tested in a mouse model. The results showed that the heater successfully delivered the therapeutic agent to the tumor site, converting radiofrequency energy into heat in the tumor cells. The mice demonstrated higher anti-tumor efficiency, prolonged survival time, and reduced tumor volume (*P* < 0. 05). The efficacy of this approach was significantly better than thermotherapy or chemotherapy alone, demonstrating that magnetic nanoparticles play a key role in delivering drugs to tumors, converting radiofrequency energy into heat in cells, and increasing apoptosis and autophagic death. Maduabuchi et al. (2023) investigated how the combination of magnetic hyperthermia and chemotherapy can selectively affect tumor growth using an *in situ* fluorescent human pancreatic cancer mouse tumor model. The team found that magnetic hyperthermia combined with chemotherapy was able to affect tumor growth and promote neoangiogenesis. The combination of magnetic induction hyperthermia and chemotherapy may be applied to transiently modulate tumor angiogenesis and improve the accuracy of drug delivery during pancreatic cancer tumor therapy.

## 6 Clinical trials

The development of magnetic materials is the key to influence the progress of MIH clinical applications. Metal heat seeds is an early application of magnetic induction hyperthermia. In 1990, researchers from Japan, Kida et al. (1990), implemented the first human clinical trial of magnetic induction hyperthermia with magnetic heat seed media worldwide. They designed an alternating magnetic field generator capable of generating a high-frequency magnetic field at 240 kHz in the center of a 30-cm-diameter induction coil and achieved a magnetic field strength of 1640 A/m. The team heated metal heat seeds made of FePt alloys to 68°C to treat 24 cases of malignant gliomas (histologically 12 glioblastomas, 10 GIII astrocytomas, and 2 GII astrocytomas). They observed degeneration of tumor cells, hemorrhage, vascular arrest and formation of thrombi found around necrotic cells. Although the results of treatment in the subcortex did not meet the researchers’ expectations, in the thalamic area, magnetic induction hyperthermia achieved favorable results (Kobayashi et al., 1990; Stea et al., 1992).

In 1992, (STEA et al., 1992; Mack et al., 1993) conducted a phase I study of mesenchymal transmissive X-ray therapy for the treatment of gliomas with the aim of testing the feasibility and toxicity of magnetic induction thermotherapy using heat seeds. The patients who participated in the test indicated that they could tolerate the entire course. The results showed that only 12 of the 28 patients survived, and the average survival of the patients was 20.6 months. Therefore, the researchers concluded that it is feasible to implant ferromagnets into brain tumors for magnetic induction hyperthermia. Subsequently, they combined thermotherapy and radiotherapy to compare it with radiotherapy alone. The results showed that the combination therapy was effective for about half of the 25 patients and could prove that magnetic induction hyperthermia was one of the factors associated with patient survival (*p* < 0.05) (Kobayashi et al., 1991).

Deger et al. (2002), Deger et al. (2004) from Germany completed a phase I and II clinical trial of magnetic induction hyperthermia combined with radiotherapy treatment for prostate cancer using alloy heat seeds in 2004. Their ongoing observation of 57 patients treated over a period of 3–72 months showed that the combination treatment was feasible and well tolerated by the patients.

Ferromagnetic heat seeds were the first magnetic material developed for magnetic induction heat therapy. Although its efficacy has been verified and many meaningful research results have been achieved, and it has been gradually used in clinic. However, ferromagnetic heat seeds are difficult to treat some irregularly shaped tumors, and are prone to uneven heating temperatures during treatment, which is not conducive to comprehensive killing of tumor cells. Therefore, the related research has to be further deepened. With the rapid progress of nanotechnology, researchers have proposed a nanoparticle-based magnetic heat therapy program. A homogeneous temperature field can be achieved by nanoscale magnetic fluids, enabling cancer-targeted therapy to advance from the tissue or organ level to the cellular level.

In 1993, Jordan’s team found that nanoscale magnetic particles have better warming properties and can achieve localized heating effects in clinically acceptable magnetic fields, and based on this, they proposed a magnetic fluid induction heating protocol known as magnetic fluid hyperthermia (MFH) (Jordan et al., 1993). In 2005, Johannsen et al. (2005b) investigated the therapeutic effect of magnetic fluid hyperthermia using a mouse prostate tumor model. The results showed that at a frequency of 100 kHz and a magnetic field strength of 18 kA/m, the maximum temperature in the tumor was 70°C or more. When the magnetic field strength was 12.6 kA/m, the average maximum and minimum temperatures inside the tumor were 54.8°C and 41.28°C. Comparing the tumor weights of the treatment and control groups, magnetic fluid hyperthermia inhibited tumor growth by 44%–51%.

In 2004, 22 patients with confirmed recurrent and residual tumors participated in a feasibility study of magnetic induction hyperthermia. These residual tumors were those that could not be resected and pre-treated, such as prostate and cervical cancers and soft tissue sarcomas. All patients received additional radiation therapy and/or chemotherapy (Wust et al., 2006). Depending on the pretreatment and location of the tumor, essentially two different techniques for the application of magnetic fluids were evaluated. One strategy consisted of CT or TRUS-guided and X-ray fluoroscopy-guided (for prostate cancer) injection, with prospective planning of magnetic fluid distribution based on three dimensional CT, MRI or ultrasound datasets. Another technique consists of intraoperative visually controlled injection.

**FIGURE 12:**
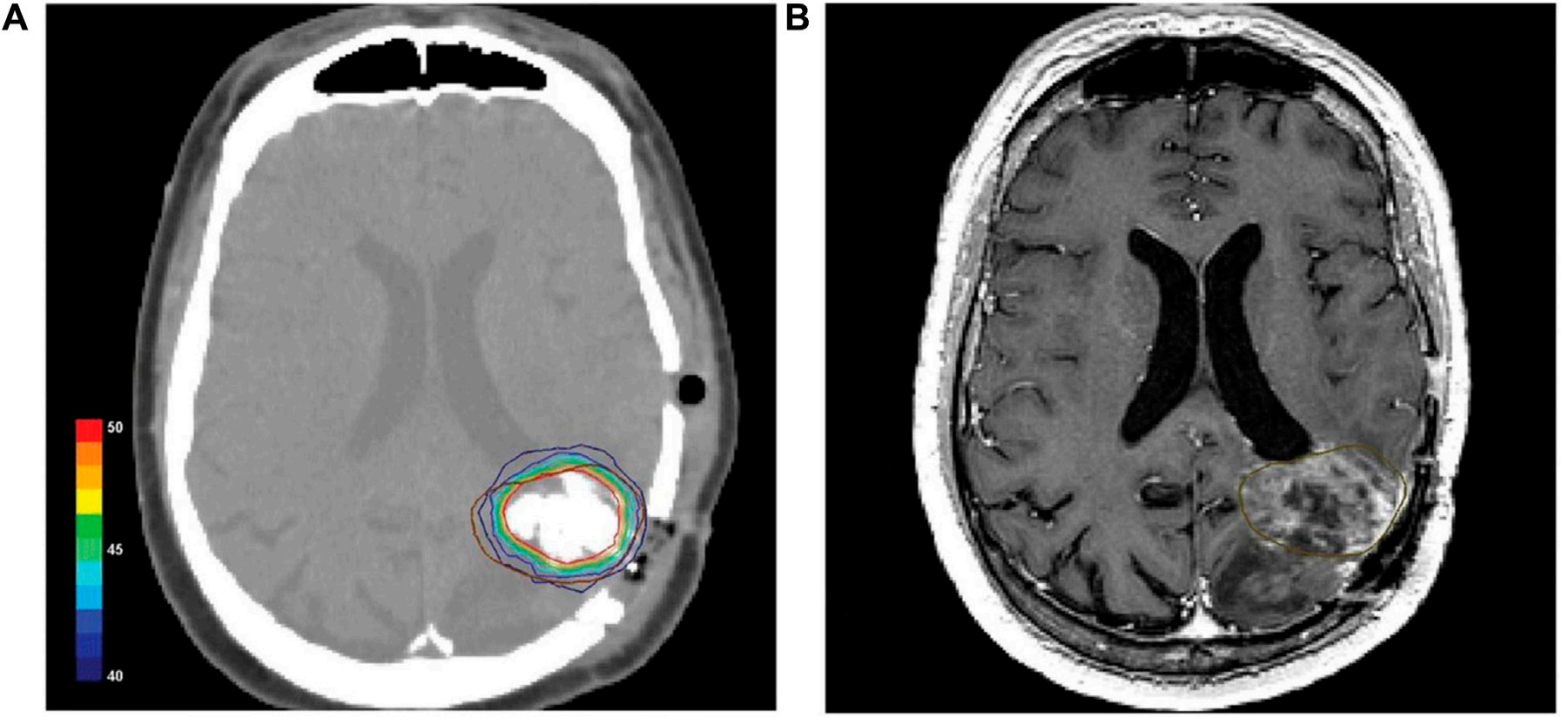
**(A)** Postoperative CT scan of a glioblastoma patient showing magnetic nanoparticles deposits as hyperdense areas within the tumor tissue. Isothermal lines indicate calculated temperatures for a magnetic field strength of 8.0 kA/m corresponding to an SAR of 718 W/kg (derived from the Hounsfield units in the CT scan). **(B)** The brown line represents the tumor area identified by preoperative MRI as fused with the CT scan (Thiesen and Jordan, 2008).

All patients tolerated the nanoparticle drops well. However, in a few cases, the pre-irradiated tumor tissue offered considerable mechanical resistance to injection and possibly also to diffusion of the magnetic fluid within the tumor. The median CT- and TRUS-guided infiltrations were 3 mL (1.5–5) and 8.5 mL (6–12.5), respectively, roughly as shown in Figure 12. Postoperative CT scan of a patient with glioblastoma showed magnetic nano-particles deposited in an area of high density (Figure 12A). The isothermal lines indicate the calculated intratumor temperature, corresponding to a magnetic field strength of 8.0 kA/m and a corresponding SAR of 718 W/kg (based on Hounsfield units in the CT scan). The brown line (Figure 12B) indicates the tumor area identified by fusion of preoperative MRI and CT scans. 0.3–0.4 mL of magnetic fluid per ml of tumor volume. Intraoperative leakage averaged 7 mL/case (2.3–10 times). Due to local discomfort (skin folds or bony surfaces), the tolerated H-field was limited to 3–5 kA/m in the pelvic region and 8.5 kA/m in the upper chest, with a median CEM_43_T_90_ of 10.5 min (range 1–106). Thermal therapy was well tolerated with only mild to moderate adverse effects (e.g., subjective heat sensation, superficial skin burns, increased pulse rate, and elevated blood pressure). Two patients (additionally implanted with 125I particles) had grade 1 to 2 perineal pain lasting up to 4 months (Thiesen and Jordan, 2008).

Ten patients with locally recurrent prostate cancer underwent a feasibility trial in the Department of Urology at the Charité University Medical School in Berlin. Magnetic fluid was injected weekly under transrectal ultrasound and fluoroscopic guidance. Patients received six weekly thermotherapy sessions lasting 60 min (Johannsen et al., 2005a). Invasive thermometry of the prostate was performed during the first and last thermotherapy sessions, and thermometry of the urinary tract and rectum was performed during each session. The maximum temperature of the prostate can reach 55°C. At field strengths of 4–5 kA/m, 90% of the prostate had a median temperature of 40.1°C (38.8–43.4), and median urethral and rectal temperatures were 40.5°C (38.4–43.6) and 39.8°C (38.2–43.4), respectively. The median thermal dose was 7.8 (range 3.5–136.4) cumulative equivalent minutes at 43°C in 90% of the prostates.

CT scans showed that the nanoparticles remained in the prostate after a 6-week treatment interval, suggesting that a single injection alone could fulfill the therapeutic needs of subsequent MIH. Nanoparticle deposition was still detectable in the prostate even after 1 year of titration. No systemic toxicity was observed at a median follow-up of 17.5 months. Acute urinary retention occurred in four patients with previous history of urethral stricture. Treatment-related morbidity was moderate, and quality of life was only mildly and temporarily impaired (Johannsen et al., 2007).

In 2007, Maier-Hauff et al. (2007) used magnetic nanoparticles to treat recurrent glioblastoma multiforme, with a total of 14 patients receiving iron oxide nanoparticle injections. The researchers exposed the patients to an alternating magnetic field to induce particle heating and found that thermotherapy using magnetic nanoparticles was well tolerated, with patients experiencing mild or no side effects. The median maximum temperature within the tumor was 44.6°C (42.4°C–49.5°C) and continued to be observed for signs of local tumor control after treatment. It was ultimately concluded that deep cranial thermotherapy using magnetic nanoparticles can be safely applied to patients with glioblastoma multiforme.

Imai et al., 2011 conducted a phase I clinical trial using MIH (Kobayashi et al., 2014). Patients with malignant tumors of the head and neck, breast, or soft tissue who had poor results with conventional treatment modalities were treated. After 2 weeks of administration, the tumors were excised under local anesthesia and pathologically examined for the therapeutic effect of MIH. The results of this clinical trial are summarized in Table 2. In all 6 patients, temperature elevations at the injection site were observed up to 43°C. No significant adverse effects were observed in this clinical trial. In pathology sections, tumor cells had necrotic changes in more than 1/3 of the tumor cells in 4 of the 6 cases.

**TABLE 2:** Summary of a Phase I clinical trial at Nagoya University Hospital, Japan (Kobayashi et al., 2014).

Cancer type	Age (years)/sex	Site	Pathological effect	Adverse effects
Papillary thyroid	79/female	Anterior neck	1a	None
Breast	52/female	Right side chest	2	None
Papillary thyroid	45/female	Left neck	0	None
Tongue	34/male	Left neck	0	None
Soft-tissue sarcoma	32/female	Right sole Right knee occipital	1b 1a	None
Follicular thyroid	55/male	Right neck (center of the target) Right neck (pheripheal zone of the target)	3 1a	None

In 2013, Jimbow et al. (2013) utilized magnetic induction thermotherapy to treat melanoma. Of the four patients with stage III and IV melanoma who received this treatment, two showed significant clinical improvement, one stage IV patient survived for 30 months with significant regression of distant skin metastases, and one stage III patient survived for more than 32 months with significant regression of local lymph node metastases. Primary tumors were excised after three local injections of MNPs conjugates to examine the local immune response to undergoing MIH therapy. Dense lymphocyte and macrophage aggregates were seen in and around the necrotic melanoma tissue.

According to a 2016 report, a patient who underwent surgical resection of a recurrent GBM tumor developed new clinical symptoms 14 weeks after receiving six 1-h sessions of MIH (Grauer et al., 2016). A CT scan of the brain showed a ring-enhancing lesion in the resection cavity surrounded by an extensive edematous area suggestive of an abscess. Surgical resection of the lesion was subsequently performed and histopathology showed persistent necrosis and heavily injected MNPs without any signs of tumor recurrence. Negative results of microbiological tests and detection of various immune cells indicated that MIH combined with radiation therapy could lead to an intense inflammatory response within the resection cavity and that the imaging features resembled an abscess.

In 2018, Grauer et al. (2019) reported the efficacy of intracavitary MIH combined with RT in the treatment of six cases of recurrent glioblastoma. Similar to the study by Maier-Hauff et al. (2011), the administration of the drug was followed by 6 sessions of 60-min magnetically induced thermotherapy. The first thermotherapy session was administered 3 days before the start of radiotherapy, followed by two sessions on the same day. All patients included in this study had previously received radiation at a median interval of 8.1 months at a standard dose of 60 Gy. During the study, four of the six patients received re-irradiation, five times per week, at a fractionated dose of 1.8 Gy, for a total dose of 39.6 Gy. Two patients were not re-irradiated because of maximum dose limitations. All patients had previously undergone surgical resection of their tumors. The mean follow-up was 11.8 ± 9.3 months. The mean survival time for patients treated for the first recurrence was 23.9 months, whereas the mean survival time for patients treated for the second recurrence and beyond was 7.1 months. Between 2 and 5 months after surgery, all patients developed significant perifocal edema around the deposits of MNPs, leading to clinical deterioration, which improved after reoperation to remove the MNPs in four of the patients. Further immunohistochemical analysis showed significant infiltration of CD3, CD8, and CD68 cells in the tumor specimens after endoluminal thermotherapy.

**FIGURE 13:**
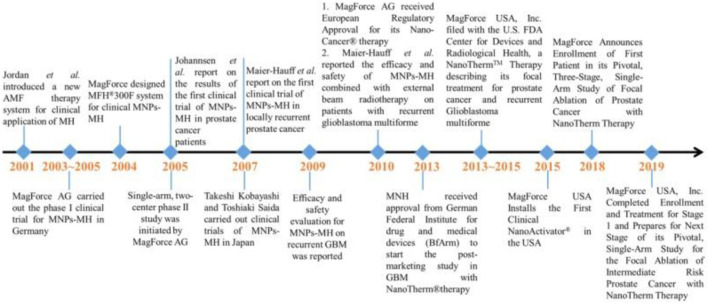
Major events associated with the clinical trials of MIH using MNPs (Liu X. et al., 2020).

In principle, MIH is capable of the non-invasive, remote regulation of cellular/subcellular activities with molecular-level specificity and without limitation in the penetration depth. Major events associated with the clinical trials of MIH using MNPs are shown in Figure 13. Based on these characteristic features, more in-depth investigative work is needed to elucidate the biological mechanisms of nanoscale heating extend the target from the single-cell level to the whole body exploited to treat cancer.

Magnetic nanoparticle hyperthermia (MNH) was approved by the European Medicines Agency for the treatment of recurrent glioblastoma in 2010 (Maier-Hauff et al., 2011) and received an Investigational Device Exemption (IDE) approval from the US FDA for prostate cancer clinical trials in 2018 (MagForce, 2019). Although researchers have conducted extensive experiments, the precise delivery of nanomedicines remains a challenge and will be the focus of the next phase of research (Kozissnik et al., 2013; Marchal et al., 2015). 2021 could be the turning point. Nantherm^®^ (for glioblastoma) has completed clinical phase 2a and the NoCanTher program has begun its final clinical trial (for advanced pancreatic ductal adenocarcinoma) (Rubia-Rodríguez et al., 2021).

In 2024, Rouni et al. (2024) developed a method for evaluating the safety of the MIH system by numerically predicting the sensible temperature rise of the skin surface layer induced by eddy current heating in the presence of an alternating magnetic field (AMF). The prediction model has been validated with clinical data obtained from a phase I feasibility clinical study in patients with stage IV solid tumors treated with standardized dose-escalating MNPs to evaluate the safety of the system. The results show that the system operates at a frequency that satisfies the limits proposed by Atkinson-Brezovich for the product of magnetic field and frequency. The numerical predictions were in good agreement with the experimental measurements, and the errors were all less than the combined uncertainty estimates, validating the accuracy of the computational model. This study highlights the critical role of simulation for translational medicine.

## 7 Alliances and distinctions between different technologies

The accuracy and efficiency of tumor treatment using magnetic induction thermotherapy alone remains challenging. By integrating multiple therapeutic modalities, it is possible to enhance the local thermal effect and improve vascular permeability within the tumor tissue, thereby amplifying drug accumulation at the target tumor site and increasing the sensitivity of tumor cells to the drug (Chang et al., 2018). Table 3 lists several examples of magnetic induction thermotherapy combined with other techniques.

**TABLE 3:** List of randomized clinical trials on hyperthermia combined with radiotherapy or/and chemotherapy.

Reference	Cancer type	Method	Number of patients randomized	Type of treatment	Outcomes	Toxicity from hyperthermia
Johannsen et al. (2005a)	Malignancy ≤3 cm in thickness from the body surface	MIH + radiotherapy	109	All patients received hyperthermia (radiative hyperthermia, 433 MHz, for ≤1 h maximum allowable temperature of normal tissue 43°C) for 1 h. If they were unable to achieve a thermal dose of ≥0.5 CEM 43°C T90, they were not randomized. Rest of patients were then randomized. Control: No further hyperthermia but had radiotherapy Experimental: Hyperthermia + radiotherapy (twice a week, 1–2 h, targeted between 10 and 100 cumulative equivalent minutes at 43°C T90)	Complete response rate: Hyperthermia arm 66% Control arm 42% *p* = 0.02 Note that some patients received systemic treatment but there was no significant difference in the proportion of patients in each arm who received systemic therapy No significant difference in overall survival	Grade 1 and 2 thermal burns 41% in experimental arm 4% in control arm Grade 3 thermal burns 5% for experimental arm 2% in control arm 11% catheter (used to monitor the temperature) related side effects for experimental arm 2% for control arm
Franckena et al. (2008)	Locoregionally advanced cervical cancer	MIH + radiotherapy	114	Control arm: Radiotherapy Experimental arm: Radiotherapy + hyperthermia (via various systems depending on site, >42°C for 60 min, 5 treatments)	12 years follow-up Local control: 37% for hyperthermia arm 56% for control *p* = 0.01	Similar rates of late toxicity between control and experimental arm
Huilgol et al. (2010)	T2-T4, N0-N3, M0 Oropharynx, hypopharynx or oral cavity carcinoma	MIH + radiotherapy	56	Control Arm: Radiotherapy Experimental Arm: Radiotherapy + hyperthermia (via capacitive system, 8.2 MHz, power increased until patients complained of discomfort, power reduced and treatment continued for 30 min, 5–7 sessions)	Statistically significant difference in median survival of control group 145 days Experimental group 241 days	Comparable acute and late toxicities between control and experimental arm, except for overall increase in thermal burns in the experimental arm
Kitamura et al. (1995)	Squamous cell carcinoma of the thoracic esophagus undergoing neoadjuvant therapy	MIH + chemotherapy	66	Control arm: Neoadjuvant chemoradiotherapy + surgery Experimental arm: Neoadjuvant hyperthermia or radiotherapy (capacitive system involving an intraluminal applicator, 42.5°C–44°C at tumor surface for 30 min, 6 sessions)	Complete response 25% in experimental arm 5.9% in control arm 3 years survival 50.4% experimental arm 24.2% control arm	Details lacking No postoperative mortality in either arm
Sneed et al. (1998)	Glioblastoma	MIH + chemotherapy	79	Control arm: Radiotherapy + oral hydroxyurea + brachytherapy boost Experimental arm: Radiotherapy + oral hydroxyurea + brachytherapy boost + hyperthermia (radiative hyperthermia, 915 MHz, ≥42.5°C for 30 min, 15–30 min before and after brachytherapy)	Median survival 76 weeks for control arm 85 weeks for hyperthermia arm *p* = 0.02	There was a trend (*p* = 0.08) toward more grade 3 or higher toxicities for the experimental arm Higher incidence of grade 1 and grade 2 neurological changes and seizures for the experimental arm
Issels et al. (2010)	Localised high-risk soft-tissue sarcoma, extremity and retroperitoneal	MIH + chemotherapy	341	Control arm: Neoadjuvant and adjuvant chemotherapy (etoposide, ifosfamide, doxorubicine) + local therapy (surgery +/radiotherapy) Experimental arm: Neoadjuvant and adjuvant chemotherapy (etoposide, ifosfamide, doxorubicine) + local therapy (surgery ± radiotherapy) + regional hyperthermia (radiative hyperthermia, 42°C for 60 min on day 1 and 4 of 3 weekly chemotherapy cycles, up to 8 sessions)	Median follow-up 34 months Significant improvement in local progression-free survival (hazard ratio = 0.58, *p* = 0.003) and disease-free survival (hazard ratio = 0.7, *p* = 0.011)	Increased pain, bolus pressure, skin burn in experimental arm

In addition to this, the researchers have combined MIH with spatial focusing. While applying an alternating magnetic field for MIH, a constant magnetic field is applied to guide the injected magnetic material. This allows for more precise heating of smaller areas (Chertok et al., 2008). Two groups of subjects were set up with static magnetic field densities of 0 T (control group) and 0.4 T (experimental group). Nanoparticles (12 mg Fe/kg) were injected intravenously to the experimental subjects, followed by 30 min of MIH. image analysis showed that magnetic targeting resulted in gliomas being exposed to a total amount of magnetic nanoparticles that was 5 times higher than that of non-targeted tumors, and that nanoparticles accumulated in gliomas with an increase of 3.6-fold in the target-selective index compared to normal brains. It can be concluded that magnetic targeting can significantly enhance the accumulation of iron oxide nanoparticles in gliomas. MIH with spatial focusing is also a promising treatment option for tumors.

It should be noted that the depth of penetration and use cases vary between technologies. Table 4 lists several different thermotherapy techniques to compare the depth of penetration and optimal application of each system.

**TABLE 4:** Different thermotherapy techniques and their applications.

Reference	Techniques	Penetration depth	Best applications
Ma et al. (2024)	Ultrasound	Less than 20 mm	Skin cancer, melanoma
Grün et al. (2013), Li et al. (2021)	Radiotherapy	50–300 mm	Breast tumor, lung cancer
Pan et al. (2021)	MIH	Excellent tissue penetration for deep-seated tumors	liver cancer, prostate cancer
Paulides et al. (2010)	Microwave	About 50 mm	Intracavitary and oral hemangiomas
Liu et al. (2020b), Guo et al. (2020)	Photothermal	Less than 20 mm	Skin cancer

## 8 Conclusion

It is evident from the discussed literature that magnetic induction hyperthermia has great potential for targeted, non-invasive tumor therapy due to its excellent therapeutic efficacy and affordability and its availability for most tumors. As an important theoretical basis for MIH, the law of electromagnetic induction and Rosensweig’s theory quantify the heating effect from millimeter-sized heat seeds to nanometer-sized magnetic fluids, and Pennes’ heat transfer equation specifically describes the heat transfer process in biological tissues, and these studies are used to evaluate the effectiveness of MIH. It is feasible to deal with some complex simulation models by using the existing computational tools, which are now reflected in the excitation coil design (Tang et al., 2023a) and temperature field analysis, helping researchers to simplify some of the preliminary work. Nanoscale magnetic fluids are the focus of research in magnetic heating media. Through the use of nanoscale magnetic fluids, the uniformity of MIH heating temperature has been substantially improved, and the trauma it produces is relatively small. As research optimizes the magneto-thermal properties of nanoscale magnetic fluids, the success rate of MIH treatments will increase in the future. The alternating magnetic fields used in MIH are poorly focused and it does not make sense to place healthy biological tissues in an alternating magnetic field. With a handheld magnetic field generator, it is possible to generate an alternating magnetic field that meets the needs of magnetically induced hyperthermia on a localized scale without creating non-essential bioelectromagnetic effects. Currently, the effectiveness of MIH in combination with chemotherapy (Shirvalilou et al., 2023) and the safety of deep MIH have been verified through experiments with rabbits, rats, and other subjects, and MIH has been approved for use in the clinical management of recurrent glioblastoma (Maier-Hauff et al., 2011).

We note that although a large number of biological experiments have been conducted, the results obtained have not been validated in clinical trials. Recently reported human trials are still ongoing, although the information is not publicly available. Whether MIH is safe and effective for different tumors in the human body will have to wait for the final results of clinical trials before a conclusion can be made. In addition, the existing research on nanomagnetic heating materials is mostly on physical properties, however, the biotoxicity of the materials is less discussed, which is insufficient to prove that the materials can be used in clinical trials. Finally, although the magnetic field focusing can be improved by improving the magnetic field generating equipment, the uniformity of the local magnetic field is still to be discussed, which is an important factor affecting the heating effect. Moreover, the magnetic field strength of the alternating magnetic field used in MIH is strong, and the safety of prolonged irradiation of the human body in this field needs to be compared with existing international standards.

At this stage, MIH is an underutilized resource in tumor therapy. Future research should aim to drive decision-making forward. For example, the coil design can be carried out by means of simulation and modeling, and the injection method of magnetic nano-fluid can be investigated on the uniformity of the temperature field, etc., which can save the preliminary workload and at the same time provide scientific guidance for the current biological experiments. At the same time, drug development should not be limited to the improvement of the magneto-thermal properties, but also the corresponding biotoxicity test should be conducted to ensure that the study is meaningful for MIH. Biological experiments are an essential part of the process. How to precisely deliver drugs into tumor tissues, and how to achieve real-time monitoring of temperature during MIH, all these important technologies for the promotion of MIH need to be verified in biological experiments.
